# PERCEPTIONS OF THE GEROPSYCHOLOGY WORKFORCE

**DOI:** 10.1093/geroni/igac059.299

**Published:** 2022-12-20

**Authors:** Jennifer Moye, Flora Ma, Nicholas Schmidt, Hannah Heintz, Rebecca Allen, Brian Carpenter

**Affiliations:** VA Boston Healthcare System, Boston, Massachusetts, United States; Stanford HealthCare Psychology, Palo Alto, California, United States; Boston VA Healthcare System, Boston, Massachusetts, United States; VA Boston Healthcare System, Jamaica Plain, Massachusetts, United States; The University of Alabama, Tuscaloosa, Alabama, United States; Washington University in St. Louis, St. Louis, Missouri, United States

## Abstract

There is a longstanding shortage of teaching faculty and clinicians trained in psychology and aging (Moye et al. 2019). Further, there are few marginalized group members in the geriatric workforce. To better understand this issue, a survey was distributed to psychology and aging listservs in preparation for the “Building Bridges” conference. Problems noted by respondents (N=275) included fewer applicants for aging-related positions (42%), decreased interest in aging by students (32%), loss of aging-related positions (18%); 24% thought workforce problems are worse than 5 years ago. Similar themes emerged in qualitative comments including: (1) lack of applicants/ interest, (2) lack of or decline in the training/ education opportunities, (3) lack of finances/ funding/ resources, (4) lack of professional positions, and (5) positive experiences/ actions/ change. Themes specific to marginalized group members to support diversity, equity, and inclusion include mindful commitment, education (e.g., mentorship), and recognizing not doing enough.

